# Bacterial lipopolysaccharide related genes signature as potential biomarker for prognosis and immune treatment in gastric cancer

**DOI:** 10.1038/s41598-023-43223-6

**Published:** 2023-09-23

**Authors:** Tianyi Yuan, Siming Zhang, Songnian He, Yijie Ma, Jianhong Chen, Jue Gu

**Affiliations:** 1 Nantong Integrated Traditional Chinese and Western Medicine Hospital, Nantong, Jiangsu China; 2 Affiliated Tumor Hospital of Nantong University, Nantong, Jiangsu China; 3 Affiliated Hospital of Nantong University, Nantong, China

**Keywords:** Bioinformatics, Genomic analysis, High-throughput screening, Risk factors, Diagnostic markers

## Abstract

The composition of microbial microenvironment is an important factor affecting the development of tumor diseases. However, due to the limitations of current technological levels, we are still unable to fully study and elucidate the depth and breadth of the impact of microorganisms on tumors, especially whether microorganisms have an impact on cancer. Therefore, the purpose of this study is to conduct in-depth research on the role and mechanism of prostate microbiome in gastric cancer (GC) based on the related genes of bacterial lipopolysaccharide (LPS) by using bioinformatics methods. Through comparison in the Toxin Genomics Database (CTD), we can find and screen out the bacterial LPS related genes. In the study, Venn plots and lasso analysis were used to obtain differentially expressed LPS related hub genes (LRHG). Afterwards, in order to establish a prognostic risk score model and column chart in LRHG features, we used univariate and multivariate Cox regression analysis for modeling and composition. In addition, we also conducted in-depth research on the clinical role of immunotherapy with TMB, MSI, KRAS mutants, and TIDE scores. We screened 9 LRHGs in the database. We constructed a prognostic risk score and column chart based on LRHG, indicating that low risk scores have a protective effect on patients. We particularly found that low risk scores are beneficial for immunotherapy through TIDE score evaluation. Based on LPS related hub genes, we established a LRHG signature, which can help predict immunotherapy and prognosis for GC patients. Bacterial lipopolysaccharide related genes can also be biomarkers to predict progression free survival in GC patients.

## Introduction

Gastric cancer (GC) is a general gastrointestinal malignancy in China. Global data show that GC ranks fifth in the incidence of all malignancies^1,2^. Environmental factors and lifestyle are the consequential causes of GC. In the past decades, the incidence of GC has gradually declined in developed countries, but it still threatens human health in developing countries. China is one of the countries with the highest incidence of GC, ranking the second in the incidence of tumors and the third in the mortality rate of malignant tumors^3^. There are 370,000 cases of death relevant to GC in China every year, even more than half of the global GC related deaths^3,4^. The backward technology of cancer screening leads to the diagnosis of most GC patients with advanced stage, accompanied by local invasion and distant metastasis, which is highly correlated with poor prognosis and mortality^5–7^. At present, gastrectomy is still the preferred treatment for patients with GC^8^. However, simple gastrectomy may not be suitable for patients with extensive invasion or lymphatic metastasis^9^. Although the pathogenesis of gastric cancer has been studied intensively by researchers around the world, the exact molecular mechanism remains unclear. Therefore, elucidating the mechanism of GC and finding effective markers of GC are of great significance for improving the prognosis of GC patients.

In addition to common infecting virus in GC development^10^, intestinal microbial has become the focus of research on GC and environmental factors, and has been widely concerned^11,12^. Under normal circumstances, the gut microbiota as a real organ associated with coordinating health and wellness of our body and the coding genes of intestinal microorganisms are 100 times higher than those of human genes^13^. Studies have pointed out that the intestinal microbiota can promote the proliferation of gastrointestinal epithelial cells^14^, ensure the normal energy and metabolism in the human body, and play an anti-inflammatory part^15,16^. Under normal circumstances, gut microbiota plays a significant part in maintaining the stability of flora and regulating the immune function of the body. After the imbalance of gastrointestinal microbiota, normal functions (immune function, metabolism, energy conversion) will be affected, resulting in the occurrence of various diseases^17–19^. The intestinal flora can affect local lesions by influencing immune factors and metabolites of the flora to have an impact on other parts of the body. With the deepening of the research on the gut microbiota, more and more studies have been conducted to diagnose the early stage of disease, treatment and prognosis by detecting microbiota markers^20–22^. As the main component of gram negative bacteria, lipopolysaccharide (LPS) plays an important role in the pathogenic components. Therefore, searching for LPS related hub genes (LRHG) has positive therapeutic significance for GC, and it is also very important to prevent the occurrence of GC.

In this study, we identified survival of GC associated with LPS relevant genes. After univariate and multivariate Cox regression analysis, we learned that LRHG could significantly predict the survival outcome of GC patients. This study examined potential molecular mechanisms of the prostate microbiome in GC. Besides, we used the chosen LRHG to construct a new prognostic model. In conclusion, our study provided a new LRHG model, and on this basis, we validated its ability in predicting the outcome of immunotherapy for GC.

## Materials and methods

### Data acquisition

**Figure 1:**
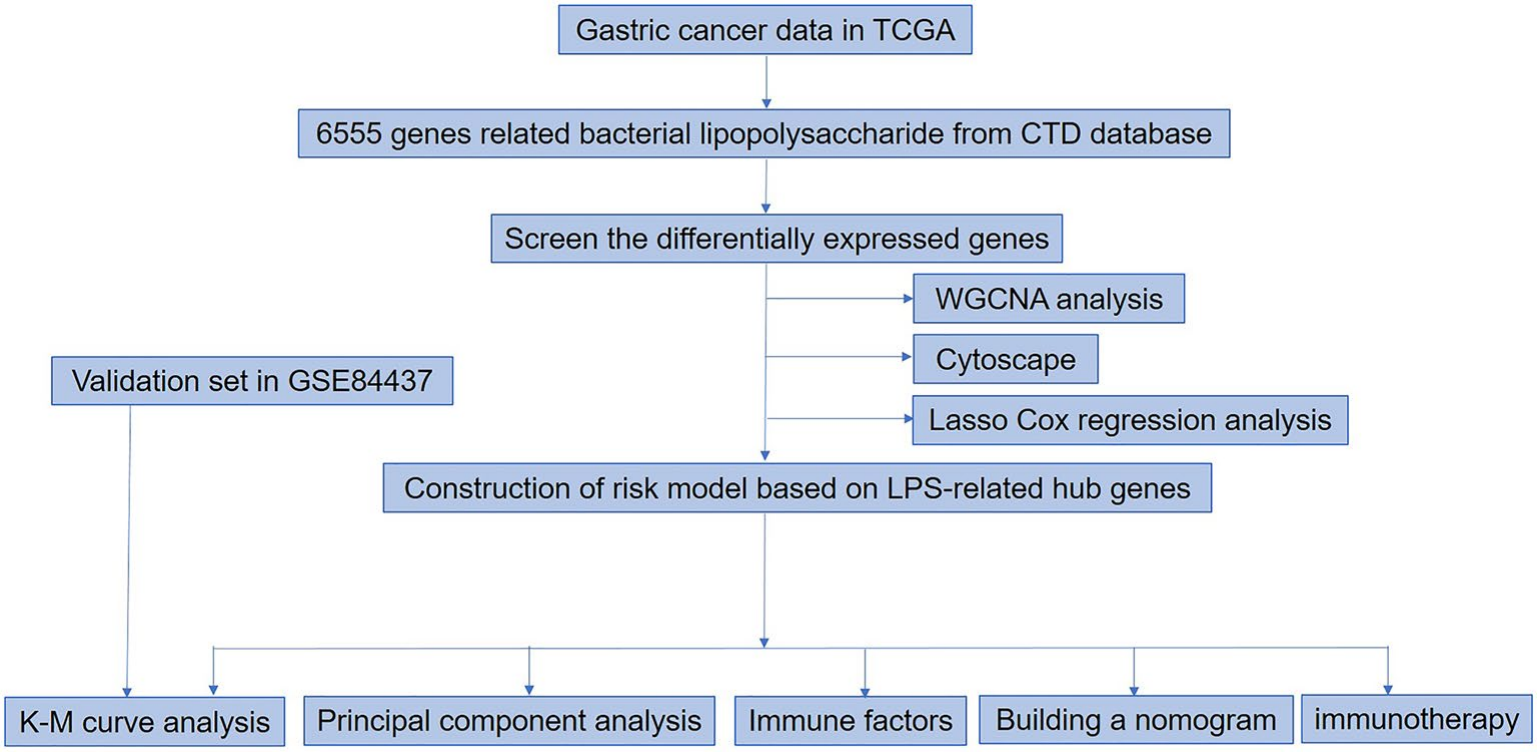
The flow chart of this study.

TCGA (https://portal.gdc.cancer.gov/) provides GC mRNA expression and clinical data with 371 patients being enrolled. 433 patients from the GSE84437 dataset were screened as the external validation set. In the process of comparative Toxicogenomics Database (CTD, http://ctdbase.org/), 6555 genes related to LPS were found. The analysis process of our study is shown in in Fig. 1.

### Gene-enrichment and functional annotation analysis

Tumor and adjacent normal’s differentially expressed genes (DEGs) defined as | logFC |> 1.5 and adjusted P < 0.05 were found. We utilize Gene ontology (GO) function enrichment and Kyoto Encyclopedia of Genes and Genomes (KEGG)^23^ pathway enrichment analyses of DEGs by R package.

### Construction of risk model based on LPS-related hub genes (LRHG)

Construct co-expression networks based on DEGs by utilizing WGCNA, which were then analyzed with clinical data using an appropriate soft-threshold power. We set the cut height to 0.3, set the minimum module size to 30, and calculate the dissimilarity results of the modules, some of which are merged. Finally, the significant positive and negative module (Blue and Turquoise) was selected for subsequent analysis. We selected prognostic genes associated with LPS based on modular genes using multivariate Cox analysis. The prediction of PPI network is input into prognostic genes in the database by using the online Search Tool for the Retrieval of Interacting Genes (STRING) database. Cytoscape tool can be used to visualize PPI networks. In the process of using plug-in cytoHubba, we comprehensively identified hub genes through Degree, DMNC, EPC, MCC and MNC scores. The ten genes with the highest degree of Degree, DMNC, EPC, MCC and MNC can be used as hub genes. Patients were divided into two groups according to LASSO cox analysis, namely, low-risk group and high-risk group. Principal Component Analysis (PCA) was applied to assess the clustering effect. To estimate the two groups' prognostic ability of the risk signature, Kaplan–Meier curves with the Log rank test were applied. To assess the performance of model predictions, the ROC curves for 1-, 3-, and 5-year survival were calculated.

### Construction of risk model nomogram based on LRHG

By establishing nomograms between risk scores and clinicopathological characteristics, we can predict 1 -, 3 -, and 5-year OS. According to the Hosmer lemeshow test, the consistency between the actual results and the predicted results can be explained by using the calculation correction curve. AUC and ROC curves were used in this study to evaluate the clinicopathological characteristics of prognosis.

### Tumor-infiltrating immune cell analysis

After CIBERSORT computation, the immune response of 22 tumor infiltrating immune cells can be estimated. Through the use of CIBERSORT R software package, the relative scores of 22 immune cell infiltration status in TCGA samples were determined. After gene set variation analysis algorithm estimation, we calculated the infiltration score of 16 immune cells and the activity of 13 immune related pathways.

### Molecular and immunological characterization of different LRHG groups and comprehensive analysis of immune treatment

As immunotherapy such as immune checkpoint inhibitor for digestive cancers is attracting increasing attention^24^,we explored the LRHG groups in different immune subtypes using the Wilcoxon test, which are based on the immune subtype profile of each TCGA sample downloaded from the UCSC Xena. Then we evaluated the TMB and MSI scores of each GC patient in the TCGA cohort. We preliminarily evaluated the widely used cancer immunotherapy biomarkers and other published immune related markers to determine the KRAS mutation of LRHG in GC patients, and then compared them with our LRHG. We can predict the prognosis of tumor patients receiving immune treatment by calculating tide score online (http://tide.dfci.harvard.edu/)^25,26^.

### Statistical analysis

R 4.2.3 (https://www.R-project.org)^27^ was used for the statistical analysis in this study. Statistics were deemed significant at P < 0.05.

## Results

### LPS-related genes and function analysis

**Figure 2:**
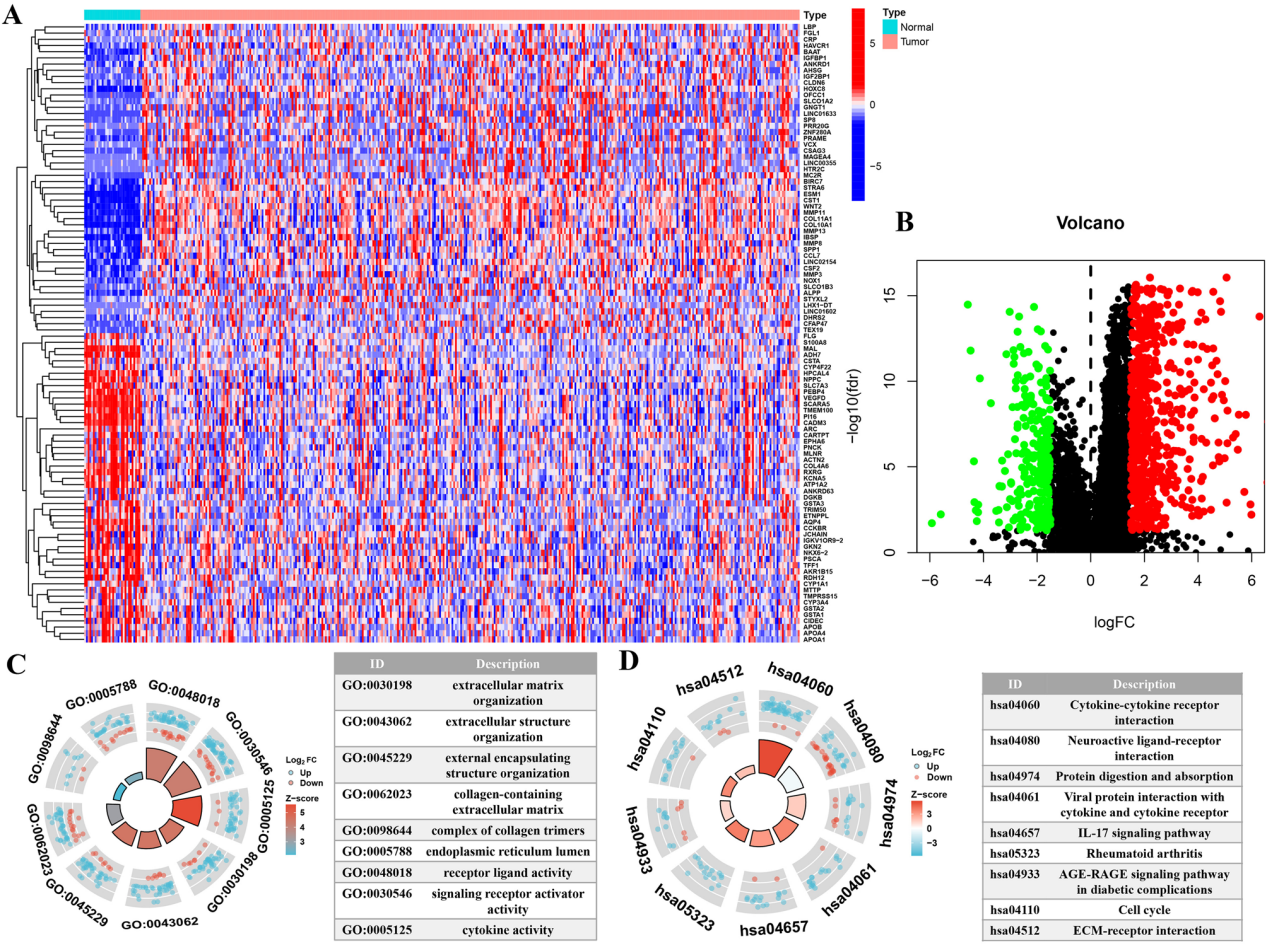
Screening for DEGs and enrichment analysis. (**A**) Heatmap of DEGs. (**B**) Volcano map of DEGs. (**C**) GO enrichment analysis for biological process, cellular component, molecular function respectively. (**D**) KEGG enrichment analysis.

In differential expression analysis, the DEGs were screened (Fig. 2A). The volcano plot showed the 853 DEGs. During the comparison between tumor samples and normal tissue samples, we found that 617 genes were up-regulated and 236 genes were down regulated (Fig. 2B). Figure 2C, D displayed the GO terms and KEGG pathways. Through functional enrichment analysis, we could see that the most relevant signal pathway of LPS-related genes was "cytokine cytokine receptor interaction". The most enriched term in biological process^28^ was molecular function^16^, and the enriched terms in cellular component (CC) were “extracellular matrix organization”, “receptor ligand activity”, and “collagen-containing extracellular matrix”, respectively.

**Figure 3:**
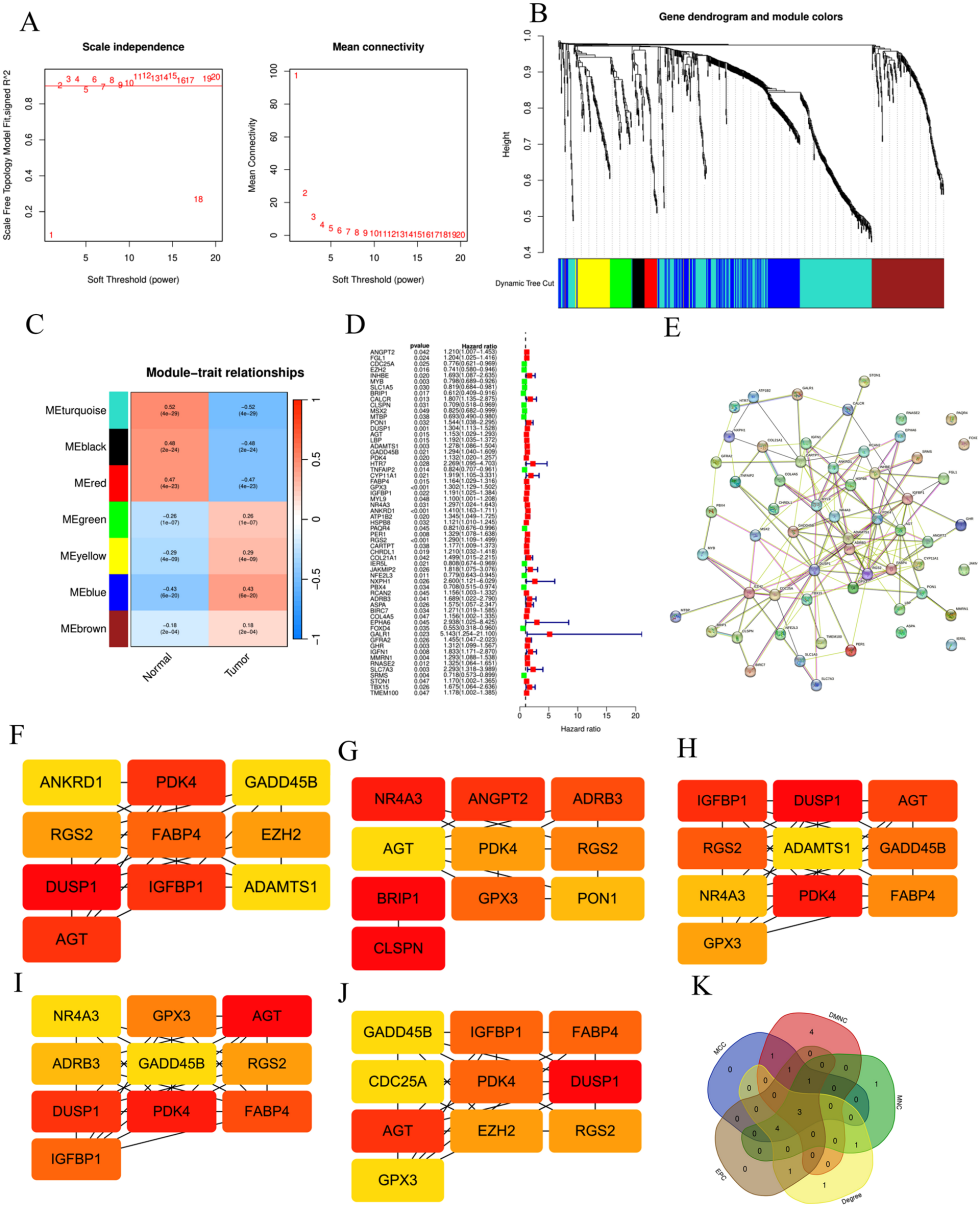
Analysis of LRHG modules by WGCNA. (**A**) Analysis of the scale-independence of various soft-thresholding powers. (**B**) Identification of co-expression modules. The branches of the tree diagram correspond to the seven different gene modules. (**C**) Correlation of gene modules with tissue type correlation scores. (**D**) Univariate Cox analysis of LRHG. (**E**) PPI network analysis of each gene in the blue and turquoise module. (**F–J**) Screen the top 10 genes by Degree, DMNC (**G**), EPC (**H**), MCC (**I**), MNC (**J**). (**K**) Veen map of Degree, DMNC, EPC, MCC and MNC.

Through WGCNA analysis of candidate genes, we extracted LPS related-genes. Through the application of scale-free network, we get that the optimal soft-thresholding power is 5 (Fig. 3A). We established a dendrogram of 853 co-expressed genes identified by DEG in the module (Fig. 3B). On this basis, we identified seven modules according to the average linkage hierarchical clustering and the optimal soft-thresholding ability (Fig. 3C). By studying the Pearson correlation coefficient between each module and the sample characteristics, we concluded that the blue and Turquoise modules were closely related to GC, and selected one of them for further analysis and research. Multivariate Cox regression analysis of OS in modular genes allows us to select independent prognostic genes for follow-up study (Fig. 3D). According to the PPI network (Fig. 3E), we chose LPS related hub genes (LRHG) by Degree (Fig. 3F), DMNC (Fig. 3G), EPC (Fig. 3H), MCC (Fig. 3I) and MNC (Fig. 3J), and united these genes (Fig. 3K).

### Survival outcomes in different LRHG groups

**Figure 4:**
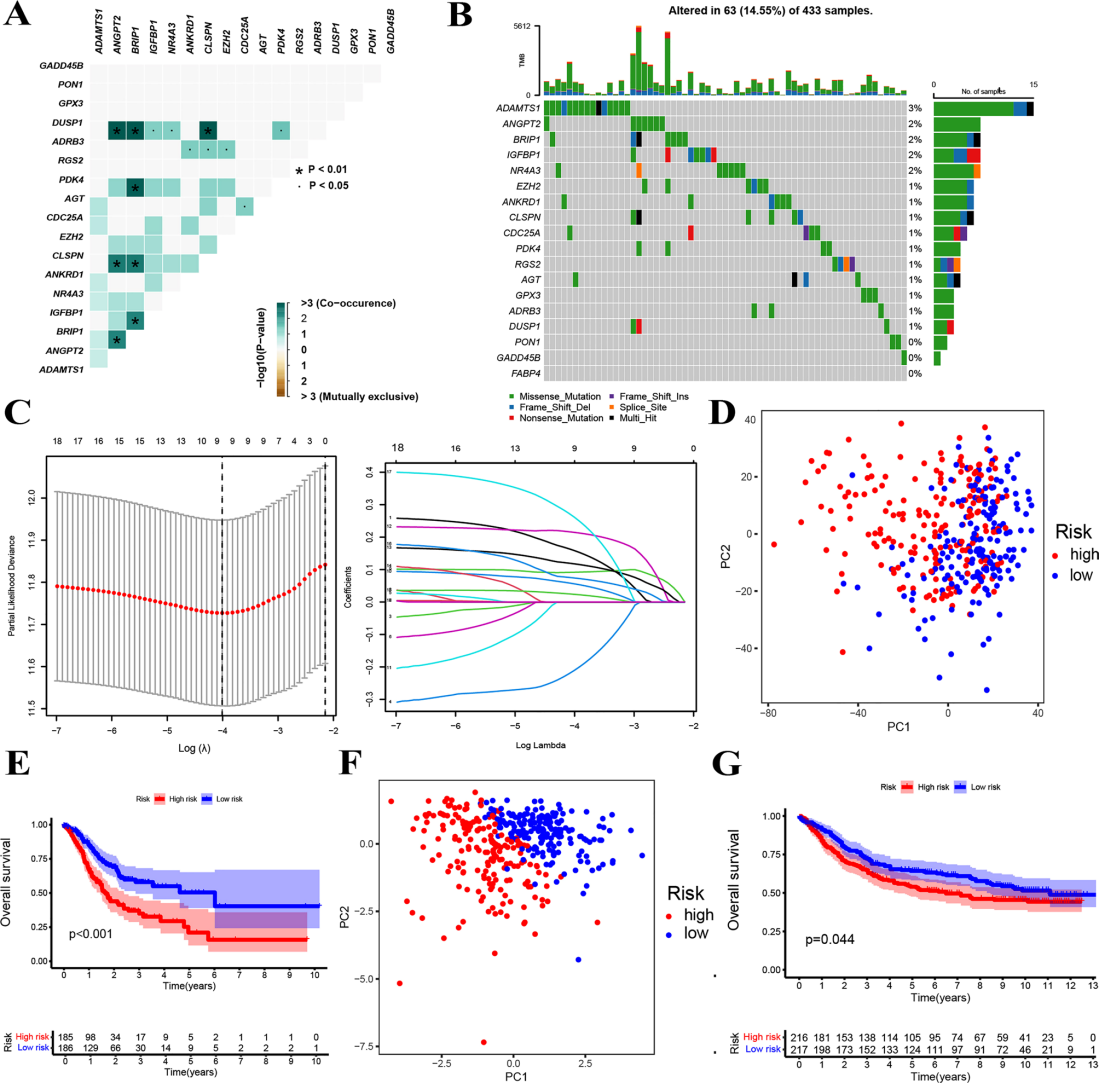
Identification of LRHG hub genes. (**A**) Correlation the LRHG genes. (**B**) Waterfall plot showing the genes mutation information. (**C**) The coefficient profile of prognostic genes by Lasso regression analysis. (**D**) PCA analysis with TCGA-STAD cohort. (**E**) Survival analysis by K–M curve for OS of TCGA-STAD patients. (**F–G**) Validation analysis in GSE84437dataset.

Firstly, we explored the association and found that DUSP1 with BRIP1, IGFBP1, NR4A3, CLSPN, PDK4 and ANGPT2 (P < 0.05); ADRB3 with ANKRD1, CLSPN and EZH2 (P < 0.01); PDK4 and BRIP1 (P < 0.05); AGT and CDC25A (P < 0.01); IGFBP1 and BRIP1 (P < 0.05); as well as CLSPN with ANGPT2 and BRIP1 (P < 0.05) had close connections (Fig. 4A). The mutant frequency of alteration between these genes displayed in Fig. 4B with DANTS1 exhibited the biggest mutation (3%), followed by ANGPT2, BRIP1, IGFBP1 and NR4A3 (2%), indicating important roles of these genes in GC development. To select independent prognostic genes, we used lasso Cox analysis to construct a prognostic index for all cancer samples (Fig. 4C), and established a risk score signature with the following formula:

$${\text{LRHG signature}}\, = \,{\text{ANGPT2}}\, * \,0.{159}\, - \,{\text{BRIP1}}\, * \,0.{211}\, + \,{\text{GPX3}}\, * \,0.0{91}\, + \,{\text{IGFBP1}}\, * \,0.0{56}\, + \,{\text{ANKRD1}}\, * \,0.{214}\, + \,{\text{RGS2}}\, * \,0.{117}\, + \,{\text{AGT}}\, * \,0.0{23}\, + \,{\text{DUSP1}}\, * \,0.0{74}\, + \,{\text{PON1}}\, * \,0.{262}.$$

In addition, we artificially divided people into two groups. PCA revealed clear boundaries between the two groups (Fig. 4D). Kaplan–Meier analysis further disclosed that the higher the risk score of TCGA GC patients, the shorter the survival time of patients (Fig. 4E). In the validation set GSE84437 similar phenomenon was also seen (Fig. 4F, G). These results revealed that constructed signature by prognostic genes of LRHG could stratify GC patients into two groups with different survival state. The inherent differences between the two groups deserve further exploration.

### Molecular characteristics of different LRHG groups

**Figure 5:**
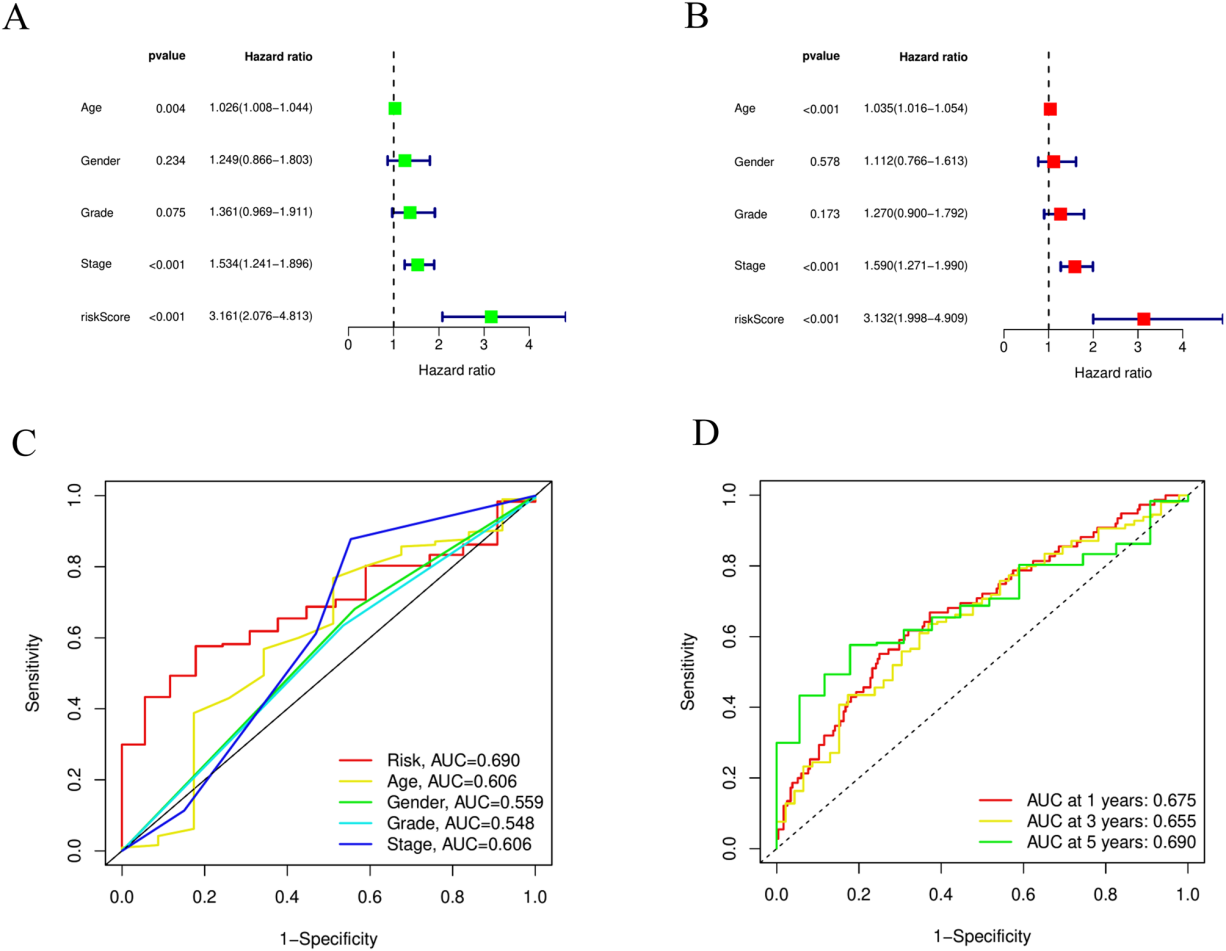
Establishing the association between the LRHG and clinical features. (**A**) Univariate and (**B**) multivariate analyses of the clinical features and risk score. (**C**) The risk score concordance indexes with clinical features in GC patients. (**D**) ROC curves in GC patients in 1-, 3-, and 5-year.

**Figure 6:**
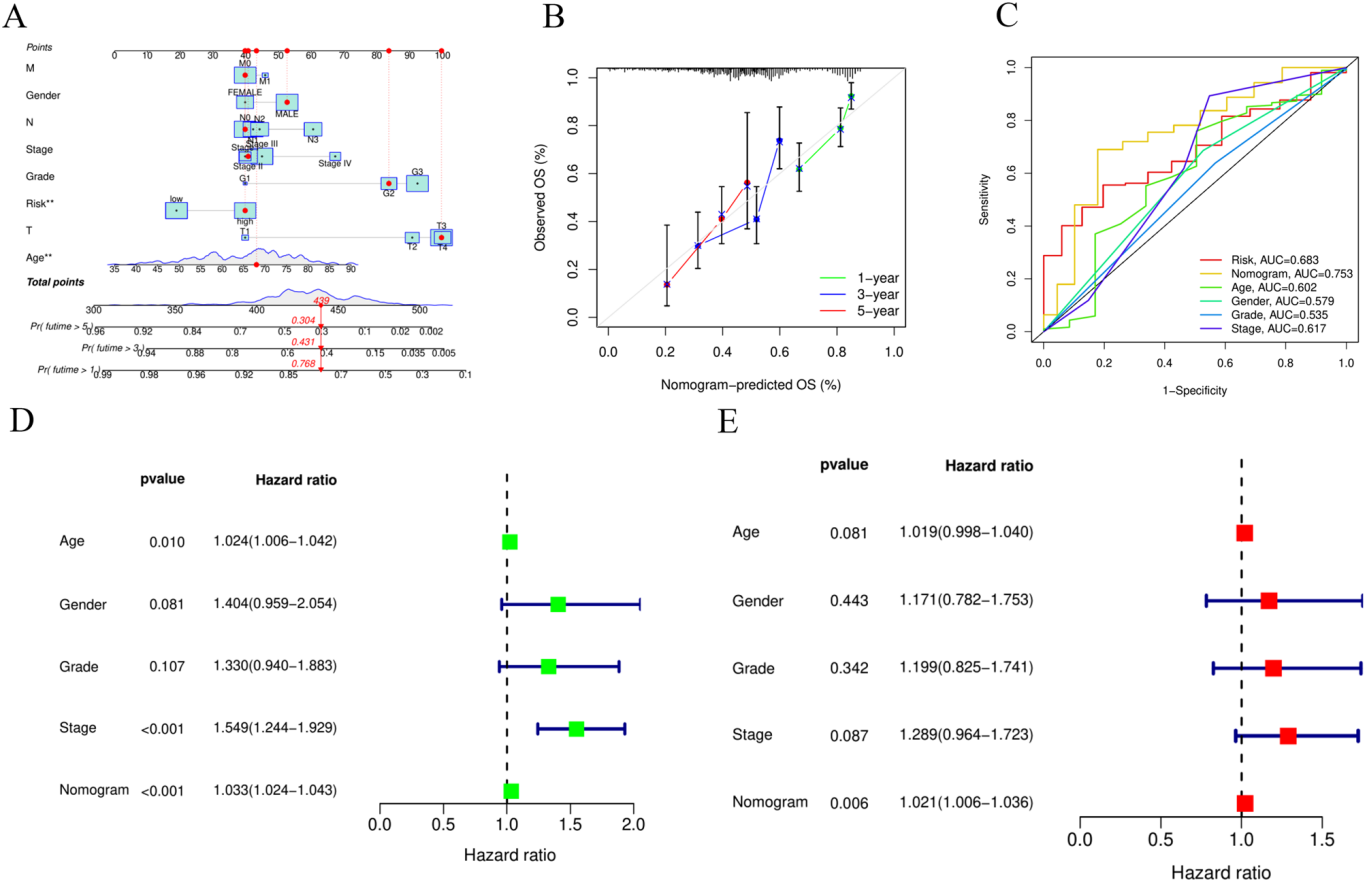
Establishing the nomogram. (**A**) The nomogram predicts the OS probability. (**B**) The calibration plot predicts the OS probability of the 1-, 3-, and 5-year. (**C**) ROC curves in GC patients with clinical features in GC patients. (**D**) Univariate and (**E**) multivariate analyses of the clinical features and nomogram.

After Cox regression analysis, based on the univariate in Fig. 5A and the multivariate in Fig. 5B, we constructed the correlation between LRHG characteristics and clinical characteristics of GC patients. From ROC curve analysis, it can be concluded that LRHG signature and clinical features are able to predict the risk, age, gender, grade and Stage with AUCs of 0.690, 0.609, 0.559, 0.548 and 0.606 (Fig. 5C). ROC curve can also show that LRHG signature to predict the 1-, 3-, and 5-year with AUCs of 0.675, 0.655, and 0.690 (Fig. 5D). Through the analysis of the predictive nomogram, the overall survival rate of the whole cohort can be predicted relatively well compared with the ideal model (Fig. 6A, B). The ROC curve showed that LRHG signature nomogram and clinical feature to predict the risk, nomogram, age, gender, grade and Stage with AUCs of 0.683, 0.753, 0.602, 0.579, 0.535 and 0.617 (Fig. 6C). In order to construct the correlation between the LRHG characteristic nomogram results and clinical characteristics of GC patients, we used the univariate Cox regression analysis in Fig. 6D and the multivariate Cox regression analysis in Fig. 6E.

### Immune characteristics of different LRHG groups

**Figure 7:**
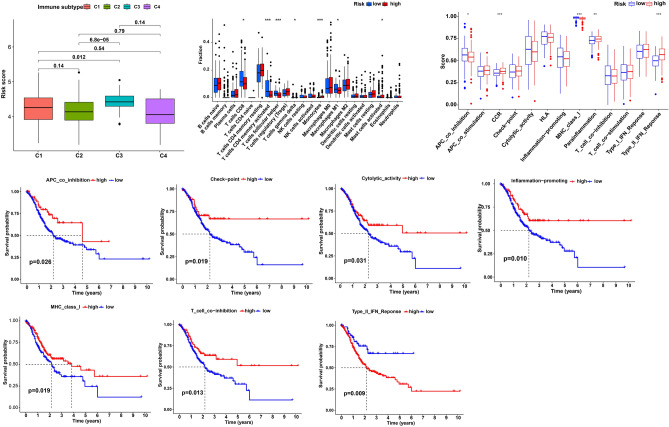
Immune cells infiltration between high-risk groups and low-risk groups. (**A**) LRHG signature analysis in different immune subtypes. (**B**) CIBERSORT showed the correlation between different groups. (**C**) Comparison of the enrichment scores of 13 immune-related pathways in LRHG group. (**D–J**) Kaplan–Meier survival analysis of the correlation of immune cell abundance ratios in the LRHG group.

Recent studies have reported that LPS induces innate immune activation^29,30^. Therefore, we explored the composition of immune cells in different LRHG prognostic indicator groups, and also used Wilcoxon test for correlation analysis. This test compared the proportion of immune cells in different LRHG prognostic index groups (Fig. 7A). We found that T cells CD8, T cells CD4 memory activated, T cells follicular helper, monocytes (P < 0.001), T cells gamma delta, macrophages M1 and eosinophils (P < 0.05) suggested differences between different LRHG prognostic indicator groups (Fig. 7B). Then, some LRHG characteristics are used to define immune and molecular functions (Fig. 7C). Through the survival analysis, GC patients which have a lower score had a better outcome, with APC co inhibition (Fig. 7D), check point (Fig. 7E), cytolytic activity (Fig. 7F), inflammation promoting (Fig. 7G), MHC class I (Fig. 7H), T cell co inhibition (Fig. 7I), Type II IFN Response (Fig. 7J).

### Benefits of immune treatment in different LRHG prognostic index groups

**Figure 8:**
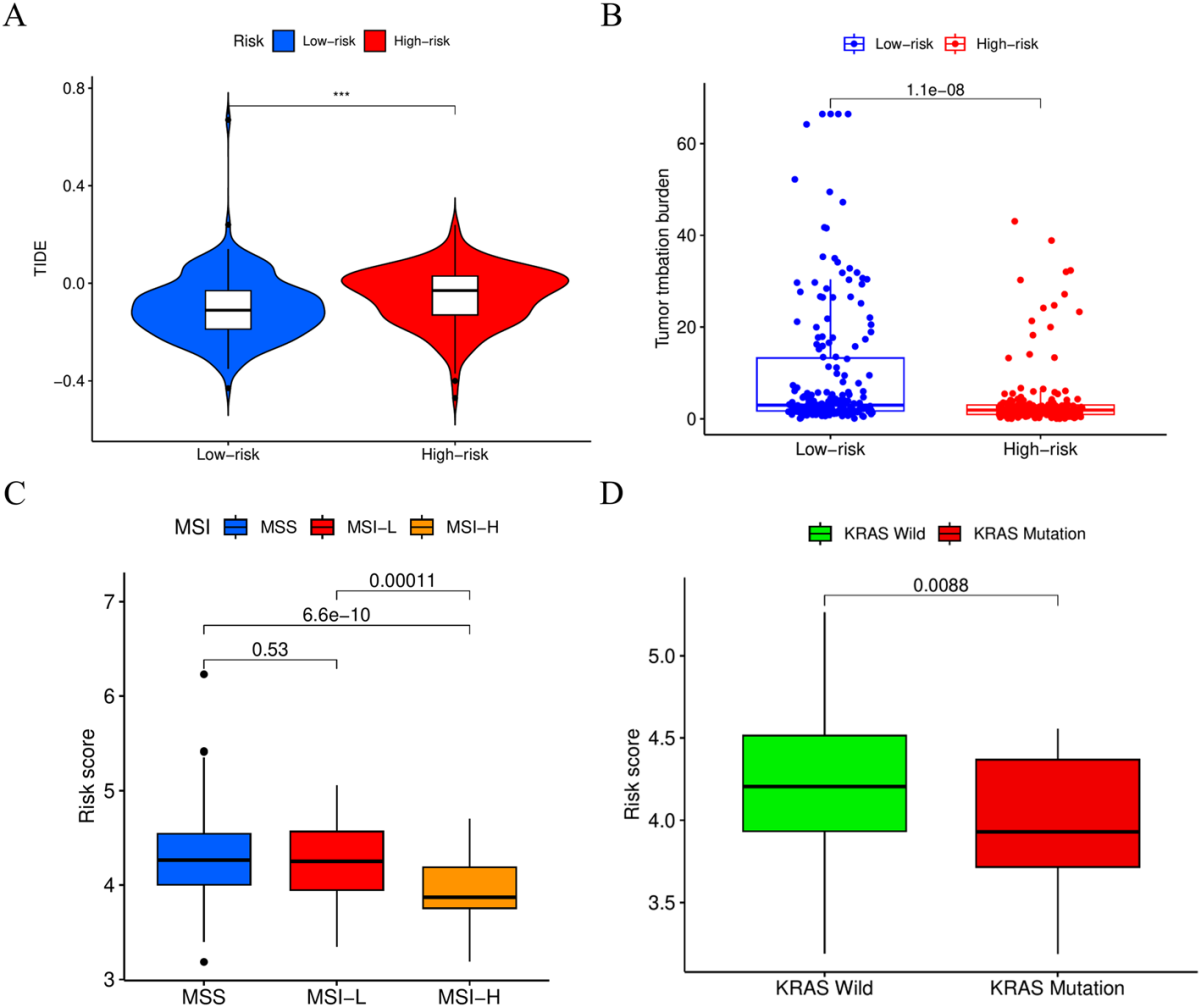
Immune response to immune therapy and the prognostic value. (**A**) TIDE score for different LRHG group. (**B**) TMB analysis, (**C**) MSI between the LRHG group. (**D**) KRAS mutant between in the LRHG high- and low-group.

The TIDE algorithm can be used to evaluate the potential efficacy of immunotherapy in different groups of LRHG prognostic indicators. The higher the tide score, the higher the possibility of immune escape, which indicates that the patients are less likely to benefit from immunotherapy^31^. In our study, we found that there were differences in TIDE score dysfunction between the two groups, which confirmed that the group with low LRHG prognosis index may be more beneficial to immunotherapy (Fig. 8A). In addition, we found that the microsatellite instability (MSI) (Fig. 8B) and tumor mutation burden (TMB) (Fig. 8C) in the low LRHG prognostic index group were lower than those in the high LRHG prognostic index group. This finding suggests that patients with low LRHG prognostic index may benefit more from immunotherapy compared to patients with high LRHG prognostic index. In recent years, KRAS mutant subpopulations might also contribute to immune therapy failure^32^. For this purpose, we explore the LRHG prognostic index-low group had less KRAS mutant compared with the LRHG prognostic index-high group (Fig. 8D). Above all, LRHG signature shows the better result, which may benefit from TMB, MSI and KRAS mutant.

## Discussion

GC is a common digestive tract tumor as a major cause of cancer-related mortality worldwide^28^. Most patients with gastric cancer are accompanied by local infiltration and distant metastasis. Currently, patients with advanced GC still have a 5-year survival rate of less than 5% after undergoing systemic treatment. So far, there is no accurate and comprehensive explanation of the exact pathogenesis of gastric cancer. To our knowledge, this is the first time that we applied widely used bioinformatics tools to explore the actions of LPS related genes on GC prognosis and immunotherapy.

Microorganisms are closely related to the development of GC, but due to the limitations of technology, the role of microorganisms in the development of GC has not been fully explained. At present, a small number of studies believe that the gastric microecology is basically unchanged in the progression of GC, and more studies believe that the gastric microorganisms is significantly changed in patients with GC. Therefore, one of the best methods may be to study and predict the role of gastric microbiome in gastric cancer patients through bioinformatics. We analyzed LPS related genes, which were obtained from the CTD database, and then combined with bioinformatics analysis to study the relevant molecular mechanisms of gastric microbiome involved in GC. Besides, we explored the immune treat in LRHG signature from TIDE score, TMB, MSI and common mutant KRAS.

For this study, the LRHG genes ANGPT2, BRIP1, GPX3, IGFBP1, ANKRD1, RGS2, AGT, DUSP1 and PON1 were obtained. In the event of bacterial LPS stimulation, silencing ANGPT2 could improve endoplasmic reticulum stress of intestinal epithelial cells via Notch signaling pathway^33^. Compared to the wild mice, RGS2 knockout mice show airways hyperreactivity and stiffer lungs in the LPS exposure^34^. Enhancing DUSP1 exerts cardioprotective effects by suppressing MAPK p38 and NF-κB pathway in LPS induced marked cardiac dysfunction model^35^. Paraoxonase 1 (PON1) was considered to have anti-inflammatory effect in previous studies^36^. Through promoting PON1 expression activated the ERK1/2 pathway, inhibiting the liver damage which is mediated by LPS^37^. However, the biological functions of LPS mediated stimulation of BRIP1, GPX3, IGFBP1, ANKRD1, and AGT are still unclear. The mechanism by which GPX3 restricts the development of colitis can be divided into two types, affecting the M2 macrophage subpopulation and promoting proliferation^38^. In addition, there are three ways for IGFBP1 to participate in tumor immunity, one is by mediating Cell surface receptor signaling pathway, and the others are by mediating cytokine production pathway or Monocyte signaling pathway^39^. In addition, AGT has the potential to serve as a biomarker for GC prognosis and immune infiltration^40^.

The research and clinical application of immune cells have pointed out new directions for cancer treatment, but patients with GC have not benefited from it. The main reason is that during the onset of GC, there is a significant immunosuppressive microenvironment, which is caused by the presence of the infection factor Helicobacter pylori, disrupting the immune balance of the gastric mucosa^36^. Therefore, we also delved into the association with LRHG and the patterns of immune infiltration in GC. The results show that there is a significant relationship between LRHG and the degree of immune cell infiltration in the tumor in T cells CD8, T cells CD4 memory activated, T cells follicular helper, monocytes, T cells gamma delta, macrophages M1 and eosinophils, especially in T cells. This study reveals that T cell CD8 can be directly activated in a manner independent of MHC class I, which has potential value for the treatment of MHC class I deficient cancers^41^.In recent studies, the completion of T cells cannot be separated from the crosstalk between T cells, intestinal cells, and other immune cells. T cells can also play an important role in the immune cell population of intestinal tissue^42^.

We investigated mutations in related genes in different LRHG groups to further understand the immunological properties of the LRHG group. We are also exploring LRHG features related to the immune microenvironment in our research. Tumor Mutation Burden (TMB) is a potential predictive indicator for cancer immunotherapy, and research has also identified limitations that hinder TMB in clinical practice^43,44^.The proportion of LRHG low group in TMB is higher than that of LRHG high group, indicating a higher correlation between LRHG low group and higher reactivity of immune checkpoint inhibitors. In addition, microsatellite instability (MSI) is the first approved pan cancer biomarker that can be used to guide the treatment of immune checkpoint inhibitors^45^. We also explored MSI and LRHG signatures during the research process. The common mutation in GC is KRAS, which plays an important role in immunotherapy for solid tumors and colorectal cancer^46,47^. Whether there is a relationship between KRAS mutations and immunotherapy in GC patients remains unproven. In our study, the tidal fraction is used to predict the probability of LRHG signature model. The analysis methods used in this process are TMB and MSI analysis. TIDE score is a newly developed prediction method to predict the response of immunotherapy. TIDE score can be more accurate than TMB or PD-L1 expression and can be used to predict the clinical effectiveness of anti-pd1 and anti-CTLA4 treatment^48^. The high tide score in the LRHG high group suggested greater immune escape and poor outcome of high LRHG score, which may be closely related to TMB, MSI and KRAS mutations.

The limitations of this study must be acknowledged, although the findings of this study provide new innovations for elucidating the molecular mechanism of GC. First, due to technical limitations, the current animal and cell experiments have no ability to comprehensively detect the composition and structural characteristics of GC microorganisms. Second, on the basis of reviewing the expression data from public databases, we identified only genes associated with LPS from CTD. Last but not least, these LRHG can indeed control GC, but the basic mechanism of their control is still not clarified, such as upstream long non-coding RNA and microRNA^49^ of these LRHG deserve to be studied. In the future, several advanced models such as integrating mathematical modeling^50^, SWATH-MS-based network modeling^51^, with experimental analysis as well as phase separation technique^52^ could be applied to explore the mechanism of our screened 9 LRHGs. What’s more, a new learning algorithm, graph convolutional network with graph attention network^53^ will also be carried out to validate our finding. In short, we need conduct comprehensive research on their biological functions through more and more rigorous techniques, algorithm and experiments. More research in the future will enable the microbiome in GC to provide diagnostic tools for GC, microbiome based prevention and immunotherapy.

## Data Availability

Publicly available datasets were analyzed in this study. These data can be found here: all relevant raw data used in the study can be accessed from TCGA (https://portal.gdc.cancer.gov/repository).
